# A Mixed Infection of Helenium Virus S With Two Distinct Isolates of Butterbur Mosaic Virus, One of Which Has a Major Deletion in an Essential Gene

**DOI:** 10.3389/fmicb.2020.612936

**Published:** 2020-12-21

**Authors:** John Hammond, Michael Reinsel, Samuel Grinstead, Ben Lockhart, Ramon Jordan, Dimitre Mollov

**Affiliations:** ^1^Floral and Nursery Plants Research Unit, United States National Arboretum, United States Department of Agriculture-Agricultural Research Service, Beltsville, MD, United States; ^2^National Germplasm Resources Laboratory, Beltsville Agricultural Research Center, United States Department of Agriculture-Agricultural Research Service, Beltsville, MD, United States; ^3^Department of Plant Pathology, University of Minnesota, St. Paul, MN, United States

**Keywords:** carlavirus, defective isolate, veronica, complementation, next-generation sequencing

## Abstract

Multiple carlaviruses infect various ornamental plants, often having limited host ranges and causing minor symptoms, yet often reducing yield or quality. In this study we have identified a mixed infection of butterbur mosaic virus (ButMV) and helenium virus S (HelVS) from a plant of veronica (*Veronica* sp.) showing foliar mosaic and distortion. Carlavirus-like particles were observed by transmission electron microscopy (TEM), and RNA from partially purified virions was amplified by random RT-PCR, yielding clones of 439–1,385 bp. Two partially overlapping clones including coat protein (CP) sequence, and two of four partial replicase clones, were closely related to ButMV-J (AB517596), previously reported only from butterbur (*Petasites japonicus*) in Japan. Two other partial replicase clones showed lower identity to multiple carlaviruses. Generic primers which amplify the 3′-terminal region of multiple carlaviruses yielded clones of three distinct sequences: (1) with 98% nt identity to HelVS; (2) ButMV-A, showing 82% nt identity to ButMV-J; and (3) ButMV-B, with 78% nt identity to each of ButMV-J and ButMV-A. Further amplification of upstream fragments revealed that ButMV-B had an internal deletion in TGB1, confirmed using isolate-specific primers. Near-complete genomes of both ButMV-A and ButMV-B were obtained from next-generation sequencing (NGS), confirming the deletion within ButMV-B, which is presumably maintained through complementation by ButMV-A. HelVS was previously reported only from *Helenium* hybrids and *Impatiens holstii*. A near-complete HelVS genome was obtained for the first time by NGS from the same sample. Additional *Veronica* hybrids infected with HelVS were identified by TEM and RT-PCR, including cv. ‘Sunny Border Blue’ which was also subjected to NGS. This resulted in assembly of an 8,615 nt near-complete HelVS genome, with high identity to that from the mixed infection. The predicted CP sequence has 96% amino acid (aa) identity to HelVS from helenium (Q00556). Other ORFs show a maximum of 54% (TGB3) to 68% (NABP) aa identity to the equivalent ORFs of other carlaviruses. These results demonstrate for the first time maintenance by complementation of a carlavirus isolate with a major deletion in an essential gene, and confirm that HelVS is a distinct species in the genus *Carlavirus*.

## Introduction

Many ornamental plants are propagated vegetatively by cuttings, or through micropropagation, in order to preserve unique characters obtained and selected through breeding, but which are not stably maintained through seed. Unless breeding populations are rigorously screened for viruses, with only virus-free seedling plants retained for propagation, this leads to the maintenance of viruses present in the original selection. Similarly, if viruses are introduced by vectors such as aphids, or by mechanical transmission due to handling of plants during horticultural operations such as transplanting, pruning, and division, this often results in mixed infections. Aphid-transmitted carlaviruses infect various ornamental plants, and often have limited host ranges; in addition, carlaviruses often produce relatively mild symptoms, which may not be identified by many growers as being due to a viral infection (e.g., Wetter and Milne, 1981). However, even viruses producing minimal symptoms in a particular host may cause more significant symptoms in a different host, or in mixed infections, and even in the absence of obvious symptoms, may reduce yield and/or quality, and ease of propagation (e.g., Brierley and Smith, 1944; Allen, 1975; Valverde et al., 2012). One classical example of a carlavirus affecting ornamentals is the case of lily symptomless virus (LSV) in Easter lily (*Lilium longiflorum*) and hybrid lilies; in single infections of most cultivars of *L. longiflorum*, LSV may cause minimal visible symptoms. However, in comparison of the growth of virus-free Asiatic hybrid lilies obtained from an LSV-infected stock, the virus-free “Enchantment” lilies were found to grow 25–50% taller than LSV-infected stock of the same cultivar, with more uniform height of the crop (Allen, 1975). The combination of LSV with cucumber mosaic virus (CMV) in a mixed infection of at least some Easter lily cultivars was found to cause necrotic stripe in the leaves (Brierley and Smith, 1944).

No carlaviruses, and few other viruses, have previously been reported from any species of *Veronica*. Impatiens necrotic spot virus was reported (as TSWV-I) in ornamental *Veronica* spp. by Hausbeck et al. (1992), while the weedy species *V. persica* has been reported to be naturally infected by CMV (Fletcher, 1989). *V. persica* was experimentally infected with Arabis mosaic virus and strawberry latent ringspot viru*s* using the nematode vector *Xiphinema diversicaudatum* (Thomas, 1970). The weeds *V. arvensis, V. agrestis*, and *V. hederifolia* are reported to be susceptible to CMV, and *V. longifolia* to alfalfa mosaic virus (Zitter, 2001), while plum pox virus has been reported on *V. hederifolia* in Bulgaria (Milusheva and Rankova, 2002).

We were therefore interested to identify the virus(es) associated with obvious mosaic in a plant of veronica submitted to the University of Minnesota Plant Disease Clinic. In this research we identified and characterized (a) a mixed infection of helenium virus S (HelVS) and two distinct isolates of butterbur mosaic virus (ButMV), one of which lacks a major portion of the “essential” TGB1 gene; (b) identified single infection of HelVS in other cultivars of veronica, established the near-complete genome of this isolate by next-generation sequencing, and confirmed it by Sanger sequencing.

Carlavirus species (family *Betaflexiviridae*, genus *Carlavirus*) are currently discriminated by the criteria of having less than about 72% nt identity or 80% aa identity, between their coat protein (CP) or polymerase genes. The complete sequence of ButMV has been available (Hashimoto et al., 2009), and clearly meets the current criteria. Both *Helenium virus S* and *Butterbur mosaic virus* are ICTV-recognized species of the genus *Carlavirus*, and while HelVS has been accepted as a distinct carlavirus species since 1982 (Matthews, 1982), to date there have been only fragments of the HelVS genome available (Foster et al., 1990; Turner et al., 1993). Indeed, to date the “RefSeq” for HelVS (NC_038323) is only a 1,389 nt 3′-proximal fragment including the CP and nucleic acid binding protein (NABP) of Foster et al. (1990).

Carlaviruses have slightly flexuous virions of c.610–700 nm long, with a single-stranded poly-adenylated genome of c.8.3–8.8 kb; the genome encodes six ORFs, with ORF1 being the viral replicase, ORFs2-4 the triple gene block, ORF5 the CP, and ORF6 an NABP (ICTV 9th Report, 2011). Previously HelVS had been reported only from Helenium hybrids (e.g., Kuschki et al., 1978) and *Impatiens* species (Koenig et al., 1983), while ButMV has been reported only from *Petasites japonicus* in Japan (Hashimoto et al., 2009) and Korea.

The results generated here represent the first near-complete genome sequence of HelVS, and demonstrate veronica as a new host for both HelVS and ButMV. Although mixed infections of different species of carlavirus have been reported previously (e.g., Van Dijk, 1993; Eastwell and Druffel, 2012; Richert-Pöggeler et al., 2015; Ho et al., 2016), and mixed isolates of other viruses in the same plant are not uncommon (e.g., Lim et al., 2010), we are not aware of any previous reports of a defective carlavirus isolate lacking an essential gene being maintained in a mixed infection with a functional isolate.

## Materials and Methods

### Plant Material

**Figure 1:**
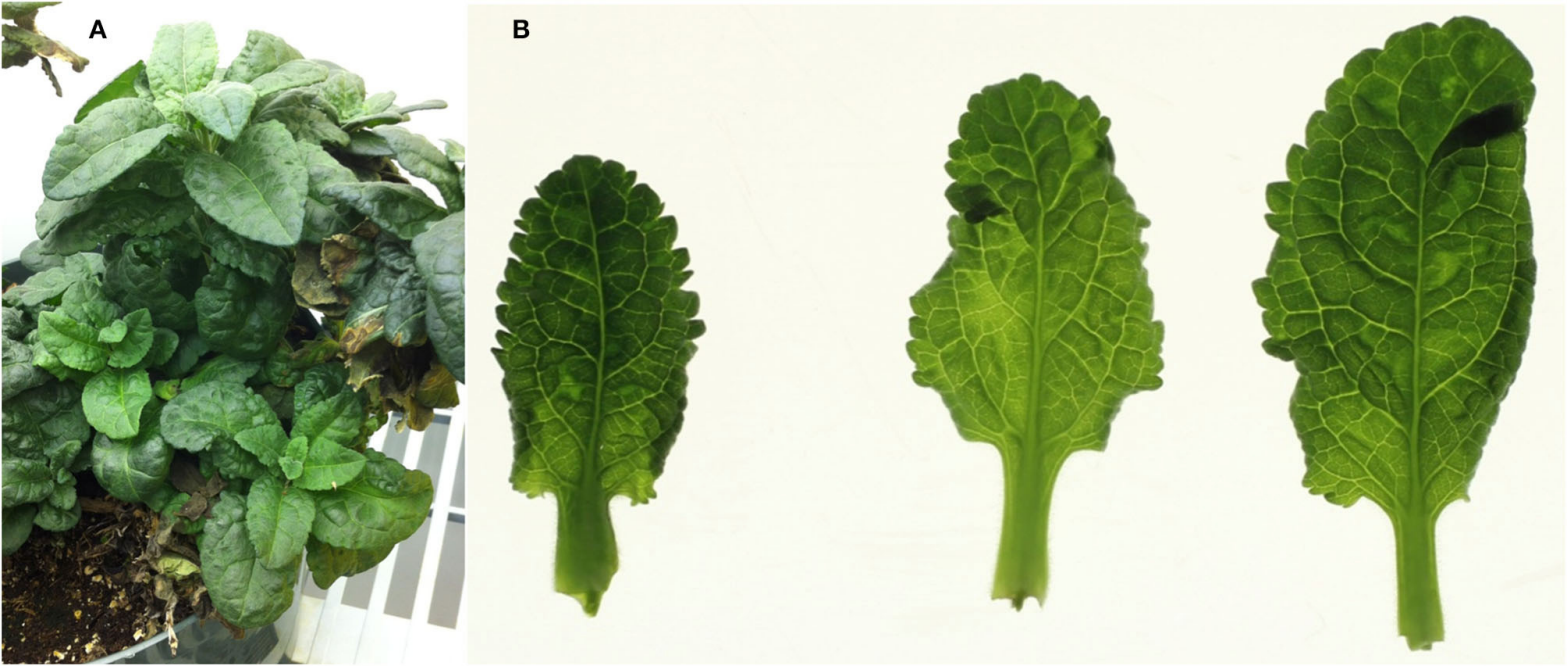
Symptoms in the whole plant, and detached leaves, of the original (Minnesota; MN) plant of *Veronica* sp., with a mixed infection of HelVS-V with ButMV-A and ButMV-B. **(A)** Whole plant showing mild foliar mosaic and puckering; and **(B)** detached leaves viewed on a lightbox, more clearly showing mottle/mosaic and interveinal chlorosis, with some puckering.

A plant of a *Veronica* hybrid showing mosaic symptoms (Figure 1) and submitted to the Plant Disease Clinic at the University of Minnesota, was the initial subject of investigation; the plant was subsequently transferred to the USDA-ARS Beltsville Agricultural Research Center, Beltsville, Maryland, where it was maintained in a growth chamber until the plant eventually died. This plant is hereafter referred to as the “MN” (Minnesota) plant. After the identification of a mixed infection in this original plant, three additional plants of *Veronica*, cultivars ‘Sunny Border Blue,’ ‘Red Fox,’ and ‘Purpleicious,’ showing mild mottle or mosaic symptoms were obtained from a local (Maryland, MD) nursery. These plants were maintained in a greenhouse under nominal day/night conditions of ~24/21°C, under natural daylight with supplemental light as necessary under cloudy conditions, and to extend day length to 14 h as required.

### Transmission Electron Microscopy (TEM)

Symptomatic leaves were initially examined at the University of Minnesota by electron microscopy. Extracts of liquid nitrogen-powdered leaves were partially purified and concentrated using ultracentrifugation through a 30% sucrose pad as described (Mollov et al., 2013). Grids were stained with 2% sodium phosphotungstic acid (PTA) pH 7.0 and visualized on a Philips electron microscope at the University of Minnesota Imaging Center. Leaf extracts of cvs. ‘Sunny Border Blue,’ ‘Red Fox,’ and ‘Purpleicious’ were prepared at USDA-ARS in Beltsville, MD, by grinding leaf pieces with a small volume of PTA and transferring a drop of the liquid to a formvar-coated copper grid for examination in a JEOL 100CX II transmission electron microscope (JEOL USA, Inc., Peabody, MA). Leaf tissue was also embedded and sectioned to examine cytopathological effects. Grids for either negatively-stained or thin sections of embedded tissue were typically examined at microscope magnifications of 33,000×, 50,000×, and 66,000×, and images captured with an AMT HR digital camera system (Advanced Microscopy Techniques Corp., Woburn, MA).

### Initial Partial Purification of Virions, cDNA Production and Cloning

At the University of Minnesota, the partially purified virion inclusive pellet was resuspended in DNase buffer and treated with DNase. Virion nucleic acids were extracted with SDS-phenol and chloroform as described (Mollov et al., 2013). A genomic library from the virion RNA was generated by random PCR technique following the protocol described by Froussard (1992). Six distinct cloned random PCR amplicons were cloned and sequenced and subjected to online BLASTn analysis at the NCBI website.

### Additional Cloning and Sequencing

Total RNA was extracted from additional leaves from the MN plant using the RNeasy Plant Mini Kit (Qiagen, Germantown, MD, USA), and cDNA was prepared using tagged oligo(dT) primer NSNC-odT (Hammond et al., 2006); PCR was performed using degenerate primer PxDeg and BNSNC, which amplify the 3′-terminal region of both potexviruses and carlaviruses (Hammond et al., 2006; Hammond and Reinsel, 2011). Additional primers were designed from the TGB2 region of the Japanese isolate of ButMV (AB517596), from the sequences of the cloned random PCR amplicons, and from the initially obtained three types of 3′-terminal region clones. Total nucleic acids (primarily RNA) was purified from leaves of *Veronica* cvs. ‘Sunny Border Blue,’ ‘Red Fox,’ and ‘Purpleicious’ using either the RNeasy Plant Mini Kit (for all isolates), and additionally the “CKC” method (Henderson and Hammond, 2013) for ‘Sunny Border Blue’ (see below), and initial cloning performed using amplicons generated with PxDeg/BNSNC. Sequences of all of the primers noted in this work are listed in Supplementary Table 1.

PCR products were cloned using the TOPO cloning kit (Invitrogen) and sequenced. Primers designed from the sequences obtained from the initial random PCR products and the 3′-terminal PCR products were used to extend the sequences and determine which upstream products were derived from the same templates as the 3′-terminal clones.

In order to verify apparent differences in size of related PCR-derived sequences derived from extension of the initial 3′-proximal PCR products, one pair of degenerate primers capable of amplifying both variants (ButRepFdeg/ButTGBRdeg) was used to amplify cDNA prepared from fresh RNA extracts from the mixed infection; two additional reverse primers, each specific for one sequence variant (ButA-TGB; and ButB-TGB) were each paired separately with ButRepFdeg for amplification, and the products analyzed by agarose gel electrophoresis.

### Library Preparation and Next-Generation Sequencing (NGS)

A total RNA extract was obtained from the original MN plant using the RNeasy kit (Qiagen). The purified RNA was submitted to Macrogen (South Korea) for cDNA library preparation and sequencing on an Illumina HiSeq2500 as 100 bp single end reads.

Total nucleic acids from the MD plant *Veronica* cv. ‘Sunny Border Blue,’ prepared using the CKC method, were submitted for ribosomal RNA depletion (RiboZero kit, Illumina) and cDNA library generation by Genewiz (South Plainfield, NJ, USA). The resulting cDNA library was sequenced in-house at USDA-ARS using 250 bp paired-end protocols on an Illumina MiSeq instrument, in parallel with other libraries, each prepared with different indices.

### Sequence Analysis

The sequences of the six initial random PCR clones were analyzed by BLASTn and BLASTp against viral sequences in GenBank. Additional sequences were similarly analyzed by BLAST, and further analyzed by local alignments, CLUSTAL alignments, and translation using tools at NCBI, and Geneious Pro or Geneious Prime (Biomatters, Inc., San Diego, CA).

### Next-Generation Sequencing

The bioinformatic pipelines utilized here were based on steps in either the CLC Workbench or Geneious packages, and are broadly comparable to other protocols that have been used by other groups. Although it has been noted that the yields of viral sequences is dependent on viral genome organization and the proportion of viral reads in the data (Pecman et al., 2017), Villamor et al. (2019) note that all of the many pipelines available share a common backbone. Massart et al. (2019) compared the ability of 21 different plant virology laboratories to detect viruses in the same 10 small RNA (21–24 nt) datasets from three different plant species, and found a high level of reproducibility between laboratories applying different pipelines to the same datasets. We therefore used the software packages available in our respective laboratories.

### Next-Generation Sequencing Analysis of the MN Sample

The 100 nt SE raw data reads were trimmed and *de novo* assembled using CLC Workbench (CLC Bio; Qiagen, USA) (8.5.1) for the ButMV analyses and version 20.0.3 for the HelVS analyses. The assembled contigs were queried against a protein database containing RefSeq virus proteins, with UniProt Arabidopsis proteins included to reduce false positive hits. Viral candidate contigs were subjected to BLASTx against the full NCBI nr database (locally downloaded to enhance speed) to refine results.

For the HelVS, data QC parameters were base caller prediction reliability *p* = 0.05 with maximum two ambiguities; assembler parameters were auto bubble and word size with minimum contig length of 175 nt. Initial BLASTx (RefSeq virus plus Arabidopsis database) parameters were BLOSUM62, gap existence cost = 11 and extension cost = 1. The second round BLASTx to the full nr database was made with the same parameters. After BLAST analyses, raw data was mapped to contigs at the criteria of 80% ID and 50% query coverage. To obtain an optimal contig length, the data was sampled at 5% to reduce coverage from up to 18,000x to no more than 900x. Further subsampling of three, two, and one percent were evaluated to obtain optimal contig length. Final assembly was at 2 and 3% subsampling. The full NGS dataset was mapped back (90% ID and 75% coverage) against this putative reassembly to correct any contig disagreements and look for any gaps or evidence of different isolates. After examining the ORFs (start codon AUG only, both strands, standard genetic code), the sequence was reverse complemented.

For the ButMV assemblies, data quality was trimmed (*p* = 0.05, maximum 2 nt ambiguity) and assembled using default auto bubble and word size, with minimum contig length 125 nt. The same BLAST algorithms were used as with the HelVS assemblies, but no subsampling was necessary.

### Next-Generation Sequencing Analysis of the MD Sample (‘Sunny Border Blue’)

The paired-end raw data reads (7,592,256 reads of 75–250 nt) from the MiSeq were binned by the specific index for the particular library, trimmed to remove adapter linkers, and *de novo* assembled into contigs using Geneious Pro R9. The Geneious Mapper parameters were set at Med-Low sensitivity and Fast assembly, 5 iterations, with 80% minimum overlap identity, allowing maximum 4 nt ambiguity, with minimum contig length 100 nt. The resulting 140,768 total contigs were analyzed by BLASTx (Blosum62, gap cost = 11, open extend = 1) against a local database of viral sequences from GenBank. Nineteen contigs (ranging in size from 1.1 kb to 5.9 kb) having high identity to carlaviruses were further edited and reassembled using the same Geneious Mapper parameters.

### Validation of the NGS Sequence From MD Plant of ‘Sunny Border Blue’

Primers (see Supplementary Table 1) designed from the assembled carlavirus-like sequences determined by NGS (HelVS-Ver, NGS) were used to generate clones of seven overlapping PCR products representing almost the full genome determined by NGS (HelVS-Ver, PCR validation). The HelVS-Ver NGS genome was also compared to the assembled c.4.1 kb PCR-derived portion of the original MN *Veronica* isolate with the mixed infection (HelVS-V); to HelVS-VR21, the NGS sequence also derived from the MN *Veronica* isolate HelVS-V; and to the partial HelVS sequences available in GenBank.

### Phylogenetic Analysis

The genomic nt coding region (including the full sequences of all recognized functional ORFs) of four virus isolates obtained in this study [HelVS-VR21 (=HelVS-V), ButMV-A, and ButMV-B isolates from the MN Veronica; and HelVS-Ver from Veronica ‘Sunny Border Blue’] were used in phylogenetic analysis with 33 carlavirus genomes obtained from NCBI. Additionally, the deduced aa sequences of the RdRp and CP ORFs for the four virus isolates were used in phylogenetic analyses with the respective carlavirus RdRp and CP aa sequences of the same 33 carlaviruses. In all cases sequences were aligned using Clustal W.

Maximum likelihood phylogenies were built using Mega X, with substitution model Tamura-Nei (Tamura and Nei, 1993), with 1,000 bootstrap replications (Kumar et al., 2018), and a condensed tree was built by collapsing branches with <50% bootstrap support.

Genome reference sequences of the carlaviruses most closely linked to HelVS in the phylogenetic trees, and identified by BLASTn, were separately compared to HelVS-VR21 using the pairwise Geneious alignment function to determine % nt identities.

### Pairwise Sequence Comparison (PASC) Analysis

The HelVS-Ver sequence was subjected to analysis using the Pairwise Sequence Comparison (PASC) tool at the NCBI website (Bao et al., 2014) to provide an addition means of verifying that HelVS is indeed a distinct species.

## Results

### Visualization of Virus Particles

**Figure 2:**
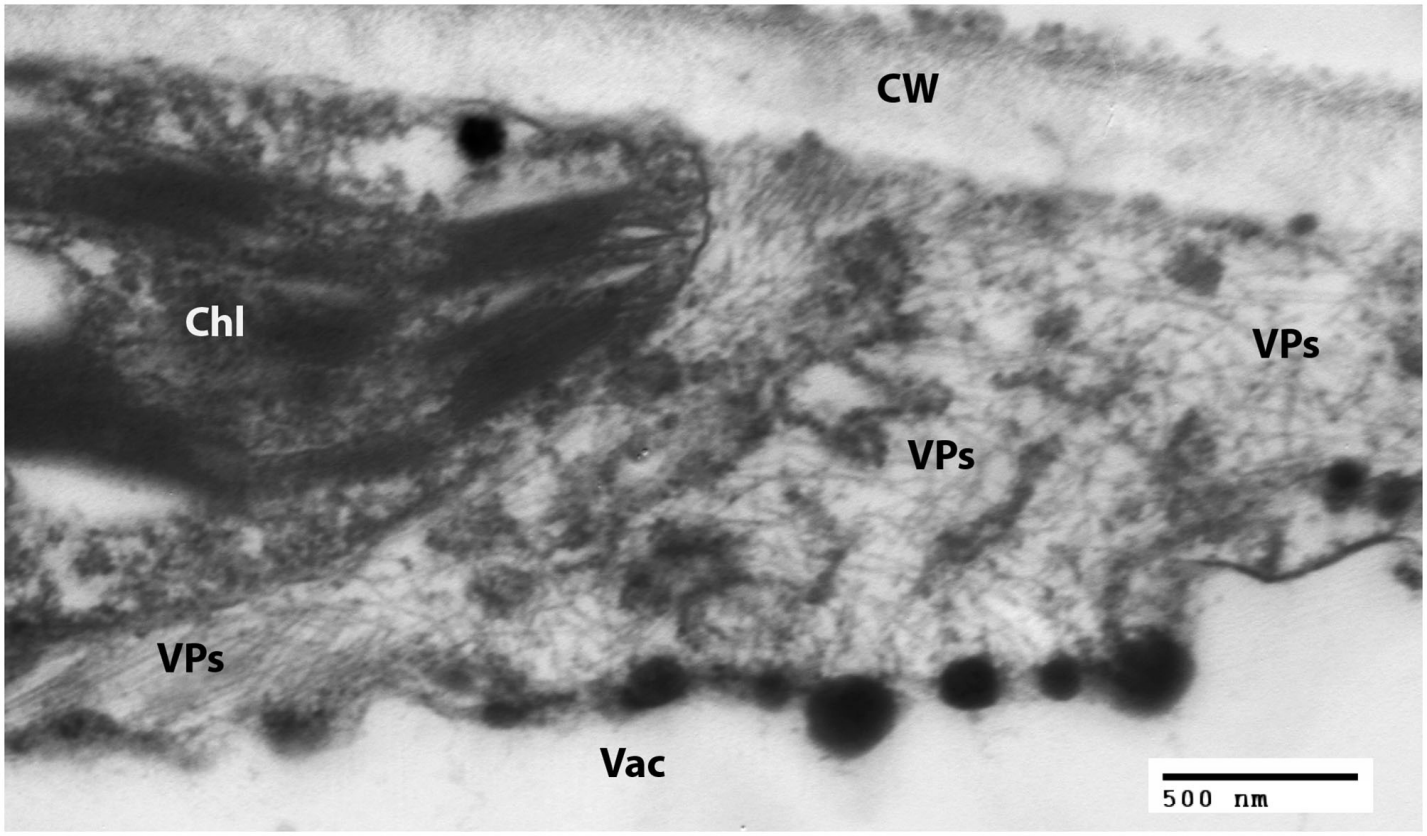
Transmission electron micrograph of a thin section of a leaf of *Veronica spicata* cv. Sunny Border Blue, showing loose aggregations of viral particles in the cytoplasm. Chl, Chloroplast; CW, Cell wall; Vac, Vacuole; VPs, Viral particles.

Examination of negatively-stained partial virus purifications at the University of Minnesota revealed slightly flexuous particles typical of carlaviruses; similar carlavirus-like virions were also detected from leaves of ‘Sunny Border Blue,’ ‘Red Fox,’ and ‘Purpleicious’ (data not shown). Examination of thin sections of embedded leaf material of ‘Sunny Border Blue’ revealed apparent slightly flexuous particles in the cytoplasm (Figure 2), similar to those observed by Koenig et al. (1983), but no banded inclusions as have been reported for various carlavirus infections by others (e.g., Wetter and Milne, 1981).

### Identification of the Origins of the Random PCR Products

The initial random PCR amplification yielded six distinct clones identified as of viral origin (Table 1); four of these clones were found to be derived from carlavirus RdRp sequences, with two partially overlapping sequences being closely related to the full genome of ButMV from Japan (ButMV-J; NC_013527) (RdRp-a, c.76%; and RdRp-c, c.80% nt identity), but distinct from each other (c.75% identity). Another partial sequence (RdRp-b) had only ~60% coverage at c.71% nt identity to ButMV-J, but 97% coverage at 73% identity to Gaillardia latent virus (GLV; KJ415259) and 94.4% identity over a 177 nt overlap to the Impatiens isolate of HelVS (HelVS-I; FJ555524); the fourth partial RdRp clone (RdRP-d) had a 100% nt identity of its 3′-end in an 86 nt overlap with the 5′-end of RdRp-b, no significant identity to ButMV-J, and higher identities with incomplete coverage to GLV (KJ415259; 65% coverage, 73% identity), Phlox virus S (PhlVS, EF492068; 49% coverage, 77% identity), Phlox virus B (PhlVB, EU162589; 51% coverage, 72% identity), and Chrysanthemum virus B (CVB, AM118099; 60% coverage, 71% identity). Two additional clones were found to have an overlap of ~450 nt in the coat protein (CP) sequence, but to share only c.78% nt identity within this overlap; the larger fragment (CP-a) included most of TGB2, all of TGB3, and most of the CP ORF, and had 79% nt identity to ButMV-J, while fragment CP-b was totally within the CP ORF, and had 82% nt identity to ButMV-J.

**Table 1:** Initial BLASTn and final identifications of origins of random PCR products.

**Random PCR product, Acc. No**.	**Size (nt)**	**Initial identification**	**Query coverage, isolate**	**Query % identity, Acc. No**.	**Final NGS sequence identification**	**% identity to NGS sequence**
RdRp-a MW226001	1,026	ButMV	99%^a^ ButMV-J	76.27 NC_013527	ButMV-B	99.81
RdRp-b MW226002	950	GLV	97% (DSM)^b^	73.43^c^ KJ415259	HelVS-V	99.58
RdRp-c MW226003	585	ButMV	99% ButMV-J	79.79 NC_013527	ButMV-A	99.49
RdRp-d MW226004	439	GLV^d^	65% (DSM)	76.21 KJ415259	HelVS-V	99.54
CP-a MW225999	1,385	ButMV	99% ButMV-J	79.23 NC_013527	ButMV-A	99.71
CP-b MW226000	775	ButMV	99% ButMV-J	81.63 NC_013527	ButMV-B	99.87

a *Pairwise alignment of RdRp-a and RdRp-c revealed 74.40% identity over the full length of RdRp-c*.

b *Deutsche Sammlung von Mikroorganismen (DSM) Gaillardia latent virus isolate 5/18-05-2010*.

c *Separate pairwise alignment with the ButMV-J sequence AB517596 revealed only 59% coverage at 71.01% identity. Pairwise BLASTn of RdRp-b against the partial RdRp sequence of the Impatiens isolate of HelVS revealed an overlap of the 3′-terminal 177 nt of RdRp-b with the 5′ FJ555524 at 92.39% identity*.

d *A pairwise BLASTn revealed no significant similarity to ButMV-J, but comparing RdRp-b and RdRP-d revealed a direct overlap (100% identity) of the 3′-terminal 86 nt of RdRp-d with the 5′-terminal region of RdRp-b, supporting their origin from the same source*.

Thus, the initial random PCR clones provided evidence for the presence of two distinct isolates of ButMV, as well as suggesting the presence of an additional species of carlavirus, initially not clearly identified.

### PCR-Based Cloning and Sequencing From the 3′ Region of the Viral Genomes

Cloning of the 3′-terminal region with either the degenerate primer PxDeg or a ButMV-specific TGB2 primer yielded three distinct sets of clones of c.1.7–1.85 kb; the sequence of these clones revealed two distinct sequences closely related to ButMV-J (designated as ButMV-A and ButMV-B), and a third sequence identified as HelVS by BLASTn analysis. We designate the HelVS sequence HelVS-V to distinguish it from the original Helenium isolate (D10454, S71594) and HelVS-I (from Impatiens; FJ555524) Further PCR amplifications were undertaken with specific forward primers designed from each of the RdRp clones, and specific reverse primers from each of the 3′-terminal clones (data not shown), or the BNSNC primer, in order to bridge the gap between the RdRp and 3′-terminal clones (Supplementary Figure 1) and generate continuous sequences of ButMV-A and ButMV-B.

This strategy resulted in generation of sets of clones enabling assembly of consensus sequences spanning 3,765 nt of ButMV-A, 3,165 nt for ButMV-B, and 3,052 nt for HelVS-V (not including the 3′ poly-A tract), with a minimum of three clones per fragment. The HelVS-V 3,052 nt sequence was then extended, by use of primer VerRdRpD-F2 designed from random PCR clone RdRp-d, paired with HVS-rep4 derived from the 3,052 nt HelVS-V sequence; after direct sequencing of the resulting PCR product using primer VerRdRpD-F1 (partially overlapping and extending downstream of VerRdRpD-F2), and sequencing the complementary strand using the PCR primer HVS-rep4. By this means the HelVS-V sequence was extended to 4,138 nt.

The HelVS-V 3′-terminal sequence was linked to RdRp-b (98.7% identity in ~153 nt overlap). The HelVS-V sequence has 98% nt identity to both available fragments of the Helenium isolate (D10545, CP and NABP; S71594, partial TGB2 and TGB3), and 96% nt identity to HelVS-I (FJ555524) (Table 2).

**Table 2:** Nucleotide identities of HeLVS sequences obtained from two Veronica sources and different methods, and compared to available GenBank accessions.

**Sequence, type, length, GenBank accession no**.	**HelVS-VR21^a^ (MN isolate), NGS, 8,668 nt, MW207172**	**HelVS-Ver (MD isolate, ‘Sunny Border Blue’), NGS, 8,615 nt, MW246808**
HelVS-VR21, NGS, 8,668 nt, MW207172		98.58%
HelVS-V^a^, PCR clone assembly, 4,138 nt, MW246812	99.76%	98.66%
HelVS-Ver, NGS, 8,615 nt, MW246808	98.58%	
HelVS-Ver, PCR validation, 8,460 nt, MW246809	98.59%	99.95%
HelVS-I, partial RdRp and TGB1, 935 nt, FJ555524^b^	94.98%	94.93%
HelVS, CP, and NABP, 1,389 nt, D10454^c^	98.13%	97.95%
HelVS, partial TGB2, TGB3, 381 nt, S71594^c^	97.64%	97.90%

a *HelVS-VR21 and HelVS-V are, respectively, the NGS-determined, and PCR-assembled, sequences of the MN isolate termed HelVS-V*.

b *HelVS-I, isolate from Impatiens, from the Netherlands*.

c *HelVS, original Helenium isolate, from the United Kingdom*.

### ButMV-B Has a Significant Deletion With Respect to ButMV-A and ButMV-J

The ButMV-A 3′-terminal sequence was linked to RdRp-c (98.7% nt identity in ~75 nt overlap), while the ButMV-B 3′-terminal sequence was linked to RdRp-a (99.4% nt identity in ~475 nt overlap). However, the ButMV-B sequence showed a significant internal deletion compared to ButMV-A and ButMV-J; the extended ButMV-A showed 81% nt identity to ButMV-J, while the ButMV-B sequence showed 72 and 77% nt identity to ButMV-J for the portions separated by the deleted sequence. ButMV-A and ButMV-B showed 71 and 78% nt identity to each other for the portions separated by the deletion in ButMV-B (Table 3).

**Table 3:** Nucleotide identities of ButMV sequences obtained from Veronica sources and different methods, and compared to available GenBank accessions.

**Sequence, length, type, GenBank Acc. no**.	**ButMV-J, 8,662 nt, RefSeq, NC_013527**	**ButMV-A, Veronica, NGS, 8,596 nt, MW207173**	**ButMV-B, Veronica, NGS, 8,033 nt, MW207174**
ButMV-J, RefSeq genome, 8,662 nt, NC_013527		77.71%	68.17%^a^
ButMV-A, Veronica, NGS genome, 8,596 nt, MW207173	77.71%		67.55%^a^
ButMV-B, Veronica, NGS genome, 8,033 nt, MW207174	68.17%^a^	67.55%^a^	
ButMV-A, Veronica, 3′ PCR clones, 3,768 nt, MW246810	81.10%	99.44%	65.97%^a^
ButMV-B, Veronica, 3′ PCR clones, 3,167 nt, MW246811	65.76%^a^	65.87%^a^	99.72%
ButMV-B, partial 5′+RdRp, 6,040 nt, (MW207174)^b^	71.75%	70.77%	
ButMV-B, TGB2, TGB3, CP, NABP-3′, 1,993 nt, (MW207174)^c^	77.12%	77.76%	
ButMV-Kr, CP gene, 973 nt, MK689358^d^	78.69%	78.59%	74.52%

a *These figures are calculated based on alignment of the complete 8,033 nt ButMV-B sequence, which has a deletion of almost the full TGB1 ORF. Because the alignment algorithms attempt to identify matches which minimize the presence of gaps, the results underestimate the nucleotide identities between the undeleted portions. We therefore separately analyzed identities to (1) the 6,040 nt region of ButMV-B containing the partial 5′-UTR, the full RdRp reading frame, and a portion of the intergenic region corresponding to that of “complete” isolates between the RdRp and TGB1 ORFs; and (2) the 1,933 nt region corresponding to the remainder of the intergenic region of “complete” isolates, plus the complete TGB2, TGB3, CP, and NABP ORFs, and the 3′-UTR*.

b *Derived from MW207174, as explained above; the 6,040 nt sequence consisting of the partial 5′-UTR, the full RdRp reading frame, and a portion of the intergenic region corresponding to that of “complete” isolates between the RdRp and TGB1 ORFs was compared against the sequences of “complete” isolates ButMV-J and ButMV-A*.

c *Derived from MW207174, as explained above; the 1,933 nt region corresponding to the remainder of the intergenic region of “complete” isolates, plus the complete TGB2, TGB3, CP, and NABP ORFs, and the 3′-UTR was compared against the sequences of “complete” isolates ButMV-J and ButMV-A*.

d *ButMV isolate Kr-NS5 from butterbur (Petasites sp.; probably P. japonicus) was detected in Korea. ButMV-J was isolated in Japan from P. japonicus, the only known natural host prior to discovery of ButMV in Veronica sp. in the USA*.

**Figure 3:**
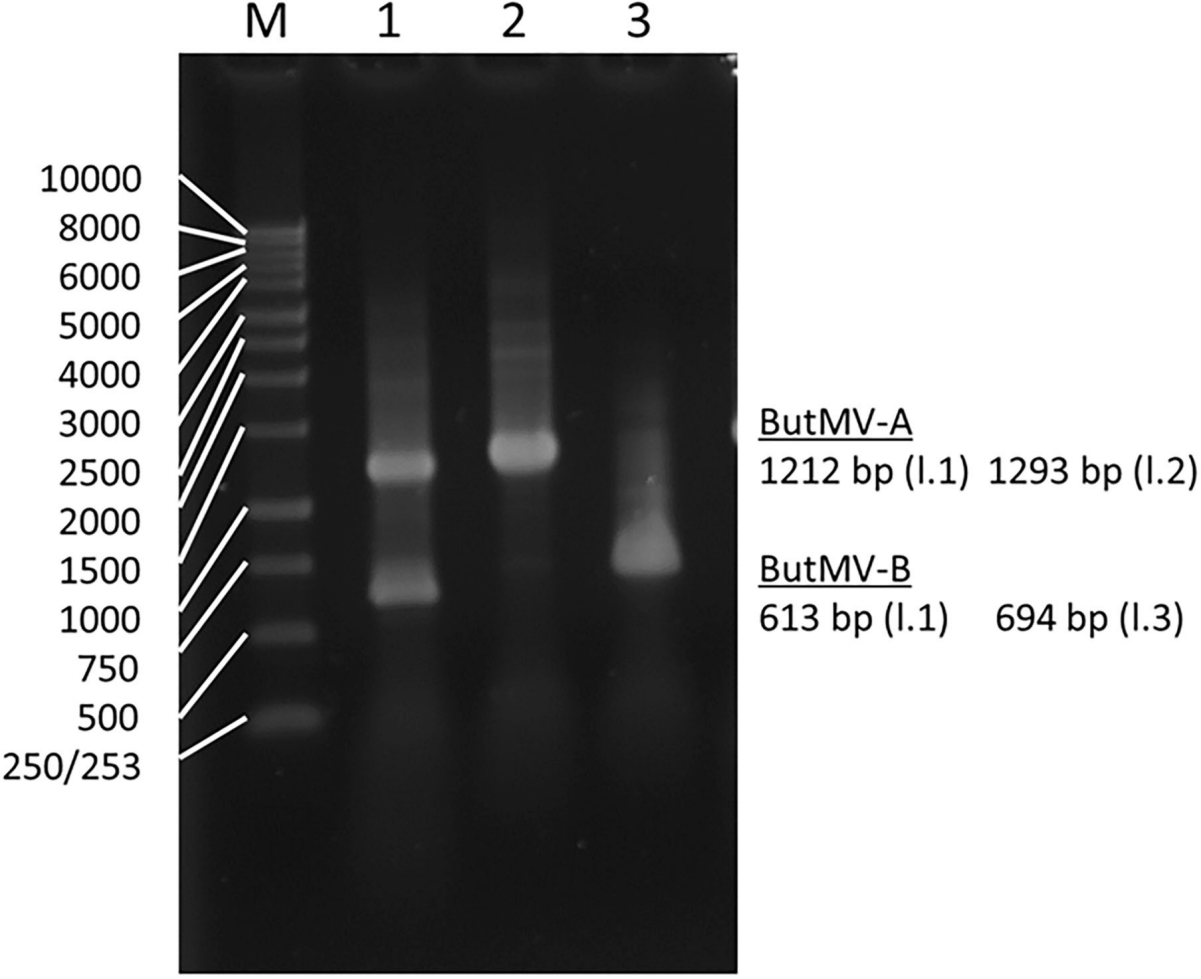
Reverse-transcription polymerase chain reaction products of assays to confirm the deletion of most of the TGB1 reading frame in ButMV-B, with RNA extracted from the original plant with the mixed infection. Lane 1—Products of degenerate primer pair ButRepFdeg/ButTGBRdeg which amplify fragments of 1,212 bp from ButMV-A, and 613 bp from ButMV-B; Lane 2—Products of ButRepFdeg paired with ButMV-A-specific reverse primer ButA-TGB, yielding an amplicon of 1,293 bp; Lane 3—Products of ButRepFdeg paired with ButMV-B-specific reverse primer ButB-TGB, yielding an amplicon of 693 bp. Lane M = 1 kb Marker ladder.

The presence of a significant deletion in the genome of ButMV-B was examined further using PCR with a degenerate forward primer designed to anneal to both ButMV-A and ButMV-B in the 3′ portion of the replicase gene, paired with: (a) ButMV isolate-specific reverse primers to span the presumed deletion; and (b) degenerate primers designed to amplify both ButMV-A and ButMV-B across the region of the deletion. Although dual PCR products corresponding to ButMV-A (1,212 bp) and ButMV-B (613 bp) were obtained with degenerate primers (ButRepFdeg/ButTGBRdeg) designed to amplify both sequences, only products representing ButMV-B sequences (with the deletion; 694 bp) or ButMV-A sequences (lacking the deletion; 1,293 bp) were recovered using the isolate-specific reverse primers (Figure 3), providing strong evidence that the deletion in the ButMV-B sequence was an authentic feature of this isolate, and not an artifact of cloning.

### NGS Results From the MN Veronica Plant

**Figure 4:**
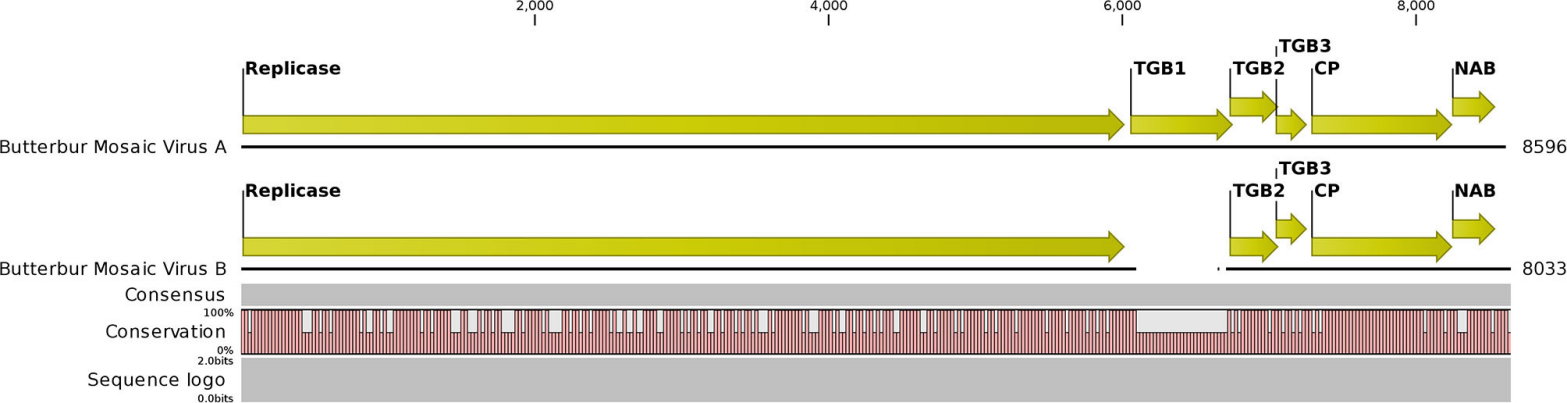
Graphical representation of an alignment of the next-generation sequencing results for the butterbur mosaic virus analysis of the mixed infection ('Conservation' line). Note gap in the ButMV-B sequence (upper), compared to ButMV-A (lower), including almost all of the TGB1 coding sequence, and compare to the ORF diagram (Top). This gap corresponds to the shorter PCR products from ButMV-B as seen in Figure 3. Degenerate forward primer ButRepFdeg is located at ButMV-A nt 5,835–5,858, and at ButMV-B nt 5,838–5,861; degenerate reverse primer ButTGBRdeg is complementary to ButMV-A nt 7,024–7,046, and ButMV-B nt 6,428–6,450. ButMV-A specific reverse primer ButA-TGB is complementary to ButMV-A nt 7,103–7,127, and ButMV-B specific reverse primer ButB-TGB is complementary to ButMV-B nt 6,507–6,531 (at equivalent positions in the aligned ButMV-A/ButMV-B genomes shown, but with multiple mis-matched positions compared to the other isolate).

The NGS run from the MN plant sample produced 29,056,208 total reads that assembled into 63,614 contigs. BLAST analysis initially revealed 31 contigs related to carlaviruses. An initial ButMV contig (later named ButMV-A) of 8,596 nt was derived from the CLC assemblies. Additionally, seven more contigs with similarities to ButMV were identified. Subsequent reassembly of these contigs merged to one consensus contig of 8,499 nt. This shorter contig (ButMV-B) had evident differences, and with further trimming and alignments, the consensus was established to be 8,033 nt long and had a significant deletion encompassing almost the full TGB1 gene when compared to the original ButMV (ButMV-A) assembly (Figure 4). Additional bioinformatics analysis produced a single HelVS contig in addition to the two distinct ButMV contigs. No significant sequences related to any other viruses were detected.

The HelVS contig was 8,668 nt (excluding the poly-A), composed of 2,001,064 reads with an average coverage of 23,072× per nt position; this sequence was designated HelVS-VR21, and represents the same HelVS isolate as the PCR-derived partial genome sequence HelVS-V. The ButMV-A isolate contig was 8,596 nt with 1,561,852 reads mapped to it and 17,773 average coverage per nt position. The isolate ButMV-B contig was 8,033 nt with 1,785,592 reads mapped to it and 21,849 average coverage per nt position.

### Comparison of Veronica Isolates ButMV-A and ButMV-B With the Type Isolate, ButMV-J, and Partial Sequence of ButMV-Kr-NS5

The NGS sequences of ButMV-A and ButMV-B were clearly consistent with data obtained by PCR amplification, showing a deletion of essentially the full TGB1 reading frame of ButMV-B (Figure 4). The portion of ButMV-B upstream of the deletion shared a lower degree of nt identity to the equivalent region of ButMV-A (70.77% nt) compared to 77.76% for the region including from TGB2 to the 3′-end of the NGS sequence; similar differences were observed for the same regions of ButMV-B compared to ButMV-J (Table 3). When sequences were compared in this manner, all of the comparisons between isolates showed >70% nt identity (Table 3). However, pairwise comparisons of each of the ORFs revealed values of c.65.7–94.9% aa identity for each ORF, with ButMV-B showing the lowest identity in all ORFs except for TGB2, which had 91.1% aa identity to that of ButMV-A; TGB3 showed the greatest diversity between isolates (c.65.7–75.7% aa identity) (Table 4).

**Table 4:** Percentage of amino acid identities between ORFs of ButMV isolates.

**Sequence**	**ButMV-J**	**ButMV-A**	**ButMV-B**
**RdRp**			
ButMV-J		85.93	79.53
ButMV-A	85.93		78.78
ButMV-B	79.53	78.78	
**TGB1**			
ButMV-J		88.05	^a^
ButMV-A	88.05		^a^
ButMV-B	^a^	^a^	
**TGB2**			
ButMV-J		86.61	86.61
ButMV-A	86.61		91.07
ButMV-B	86.61	91.07	
**TGB3**			
ButMV-J		75.71	65.71
ButMV-A	75.71		67.14
ButMV-B	65.71	67.14	
**CP**			
ButMV-J		91.10	88.65
ButMV-A	91.10		86.81
ButMV-B	88.65	86.81	
**NABP**			
ButMV-J		94.85	85.71
ButMV-A	94.85		83.51
ButMV-B	85.71	83.51	

a *ButMV-B has a deletion of almost the entire TGB1 reading frame, leaving only a portion of ~14 codons, of which seven codons overlap with the TGB2 initiation codon and reading frame. There are five termination codons downstream of the ButMV-B RdRP termination codon, and in frame with the remaining fragment of the TGB1 reading frame*.

The TGB1 ORF (missing in ButMV-B) of ButMV-A and ButMV-J each had a potential TTG alternate initiation codon in identical context (GTT.AGT.**TTG**.AAA.TAT.**ATG**.GAC) three codons upstream of the presumed native start codon, which would result in addition of three N-terminal residues (MKY). TGB2 of ButMV-A and ButMV-J each had CTG in a slightly different context (TCC.TTA.**CTG**.GAG.**ATG**.CCT for ButMV-A; TTC.TGA.**CTG**.GAG.**ATG**.CCT for ButMV-J), but in each case encoding two additional residues (ME); ButMV-B had no potential alternate initiation codon. The CP ORFs of each isolate had potential TTG starts in similar contexts, which would add different combinations of seven or eight additional residues (GTT.TGA.**TTG**.AAT.ACG.AAT.TCC.CAA.AAA.CAT.**ATG**.GGG, MNTNSAKH for ButMV-A; TTT.TGA.**TTG**.AAT.AGT.TTT.TCC.AAA.CAA.**ATG**.GGT, MNSFSKQ for ButMV-B; TTT.TGA.**TTG**.AAT.ACG.AAT.TCC.CAA.AAA.CAT.**ATG**.GGG, MNTNSAKH for ButMV-J). There was a potential CTG initiation codon immediately upstream of the native ATG in the NABP ORFs of ButMV-B and ButMV-J in slightly different context (CTC.GGA.**CTG**.**ATG**.TCA for ButMV-B; CTC.TGA.**CTG**.**ATG**.TCG for ButMV-B) adding only an additional Met residue, but not for ButMV-A. Note that in all three isolates the native NABP ATG overlaps the termination codon of the CP ORF in the (−1) reading frame.

### NGS Results From Veronica ‘Sunny Border Blue’

The raw reads (7,592,256 reads of 75–250 nt) were assembled into contigs, analyzed by BLASTx, and those contigs having high identity to carlaviruses were further edited and assembled into a 8,615 nt near-complete sequence of the isolate named HelVS-Ver. The mean coverage per nt position was 14,493 reads with 892,791 total reads mapping to the consensus sequence. There was significant coverage across all regions of the genome except the extreme 5′ and 3′ regions (Supplementary Figure 2). The HelVS-Ver sequence lacks an estimated 41–70 nt of the 5′ UTR (by comparison to the length of HelVS-VR21, and of carnation latent virus, respectively; this report, and R. Jordan, D. Mollov, and S. Grinstead, unpublished), and 25 nt of the 3′-UTR (by comparison to previously determined HelVS sequences from veronica, including the 3′-terminal clones developed here). Primers designed from the NGS-derived genomic sequence were used to generate seven over-lapping amplicons extending to the 3′ poly(A), which were cloned and sequenced to validate the NGS-derived genome; a minimum of five clones representing each of these overlapping PCR products were Sanger sequenced in both directions, and the consensus sequences assembled to create a validated sequence representing 8,464 bp (Supplementary Figure 3).

### Comparison of HelVS Sequences HelVS-Ver, HelVS-VR21, and Partial Sequence of Helenium and Impatiens Isolates

The NGS genomes [HelVS-Ver and HelVS-VR21 (from isolate HelVS-V)] were also compared to the 4.1 kb sequence previously obtained by PCR extension from the MN veronica isolate HelVS-V. The NGS 8,615 nt sequence of HelVS-Ver and the 8,464 nt PCR-validated sequence showed 99.95% nt identity, while the NGS sequences from the MN isolate HelVS-V and HelVS-Ver shared 98.58% identity over the length of the HelVS-Ver sequence (Table 2). The near-complete NGS sequence of HelVS-V (=HelVS-VR21) and the PCR extension sequence of 4,138 nt (HelVS-V) from the same plant shared 99.76% nt identity, similar to the degree of identity between the NGS and PCR-validated sequence of HelVS-Ver (Table 2). The full genome and 3′-UTR nt sequences of HelVS-Ver and HelVS-VR21, and their predicted aa sequences of each of the ORFs, all shared a minimum 98.58% identity, with 100% identity for the TGB3 and NABP ORFs (Table 5).

**Table 5:** Percentage of nucleotide (nt) and amino acid (aa) identities between HelVS isolates V (HelVS-VR21) and Ver (HelVS-Ver).

	**HelVS-VR21**							
**Region**	**Genome nt**	**RdRp** **aa**	**TGB1 aa**	**TGB2** **aa**	**TGB3 aa**	**CP** **aa**	**NABP aa**	**3′** **UTR** **nt**
HelVS-Ver	98.58	98.81	99.13	99.07	100	99.0	100	98.67

Interestingly, both the TGB1 and TGB3 ORFs of HelVS-Ver and HelVS-V had potential TTG alternative start sites upstream of the presumed native ATG codon, which would, if utilized, add the same N-terminal aa residues: MF in the case of TGB1; and MWPASL for TGB3. The sequence context for the potential alternate start codons for both isolates was shared for TGB1 (GTG.AAA.**TTG**.TTT. **ATG**.GAT), and also shared for TGB3 (GTG.TAG.**TTG**.TGG.CCG.GCG.TCA.TTA.**ATG**.TCA).

### Pairwise Sequence Comparison (PASC) Confirms That HelVS Is a Distinct Carlavirus Species

The sequence of HelVS-Ver was compared to other carlavirus genome sequences using the Pairwise Sequence Comparison (PASC) tool at the NCBI website (Bao et al., 2014). This analysis clearly identified HelVS as a distinct carlavirus sequence, most closely related to GLV, PhlVS, and Ligustrum virus A (LVA) (Supplementary Figure 4).

### Phylogenetic Analysis

The near-complete genome nt sequences of HelVS-VR21 and HelVS-Ver, ButMV-A and ButMV-B, plus the complete deduced aa sequences of their RdRp and CP proteins were used to generate Maximum Likelihood trees together with the equivalent sequences of multiple other ICTV-recognized carlavirus species.

**Figure 5:**
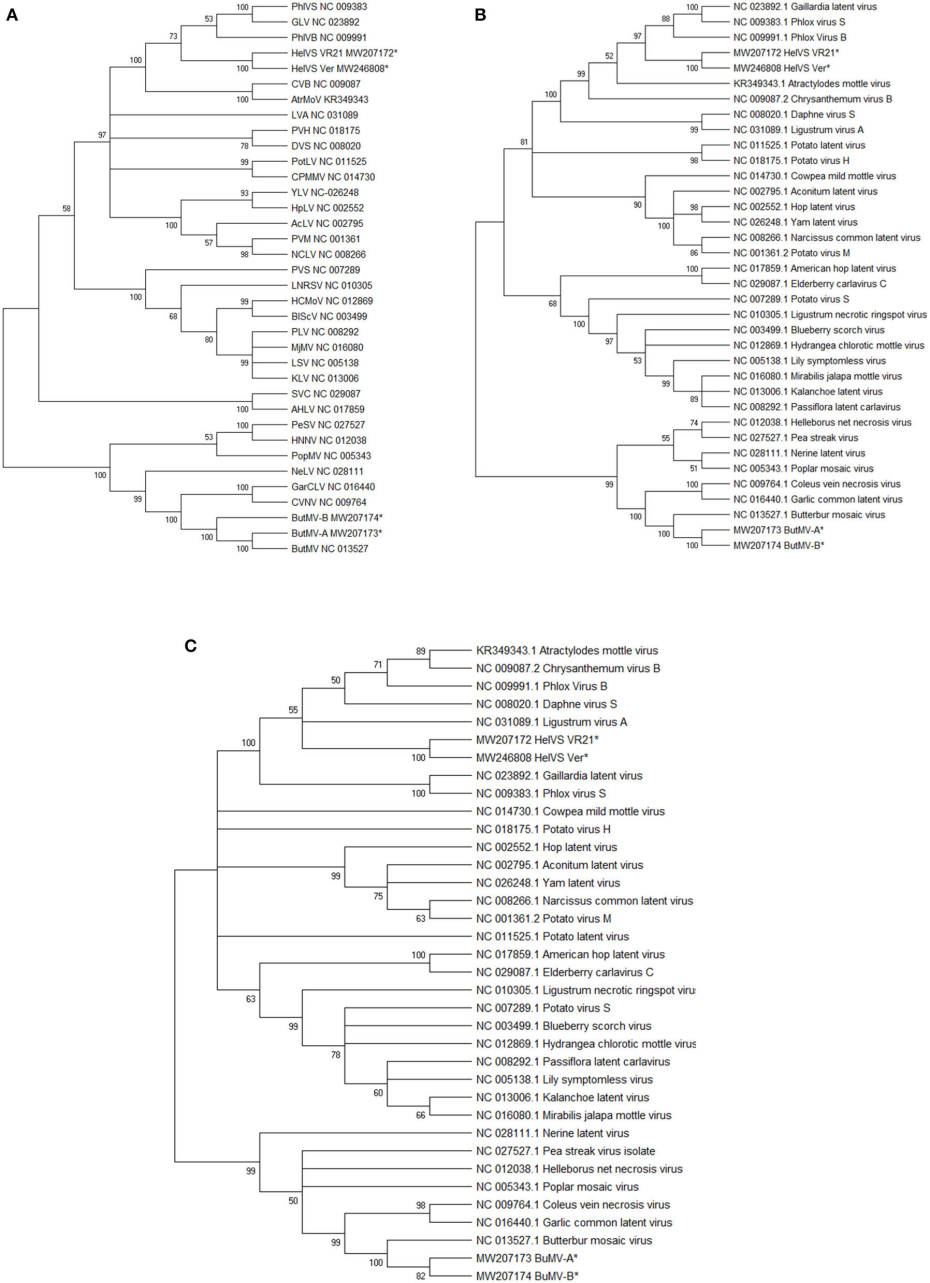
**(A–C)** Maximum Likelihood phylogenetic trees with 1,000 bootstrap replications of: **(A)**, the genomic nucleotide sequences; **(B)** the Replicase (RdRp) amino acid sequences; and **(C)** the Coat protein (CP) amino acid sequences. Branches with <50% bootstrap support were collapsed. The GenBank accession number for the genomes are listed following the virus acronym in **(A)**; the RdRp and CP sequences were taken from the genome accessions. Virus abbreviations: AcLV, Aconitum latent virus; AHLV, American hop latent virus; AtrMoV, Atractylodes mottle virus; BlScV, blueberry scorch virus; ButMV, butterbur mosaic virus; CPMMV, cowpea mild mottle virus; CVB, chrysanthemum virus B; CVNV, coleus vein necrosis virus; DVS, Daphne virus S; GarCLV, garlic common latent virus; GLV, Gaillardia latent virus; HCMoV, hydrangea chlorotic mottle virus; HelVS, Helenium virus S; HNNV, Helleborus net necrosis virus; HpLV, hop latent virus; KLV, kalanchoe latent virus; LNRSV, Ligustrum necrotic ringspot virus; LSV, lily symptomless virus; LVA, Ligustrum virus A; MjMV, Mirabilis jalapa mottle virus; NCLV, narcissus common latent virus; NeLV, Nerine latent virus; PeSV, pea streak virus; PhlVB, phlox virus B; PhlVS, phlox virus S; PLV, Passiflora latent virus; PopMV, poplar mosaic virus; PotLV, potato latent virus; PVH, potato virus H; PVM, potato virus M; PVS, potato virus S; SVC, Sambucus virus C; YLV, Yam latent virus. The next-generation sequences of both isolates of HelVS, and both isolates of ButMV from the current studies are included in each tree, and each is marked with an asterisk (^*^).

Each of these trees showed broadly similar topology, with the two HelVS isolates grouped in a clade including PhlVB, PhlVS, GLV, CVB, and Atractylodes mottle virus (AtrMoV), with 100% bootstrap support; LVA and daphne virus S were also within the clade for the RdRp and CP trees, with 100% bootstrap support (Figures 5A–C).

BLASTn searches with the HelVS-VR21 genome yielded high identity to the HelVS CP and NABP (NC_038323), and the HelVS partial replicase/TGB1 (FJ555524); the genome of GLV showed 70.60% nt identity, but only 53% coverage, while all matches to other viruses had low identities and coverage. BLASTp searches of the HelVS-VR21 RdRp yielded no more than 55.93% aa identity to multiple carlaviruses, while BLASTp analysis of the HeVS-VR21 CP identified 96.99% aa identity to HelVS CP (YP_009505624); <72% aa identity (83% coverage) to red clover carlavirus A (AUF71594); and <66% aa identity to other carlavirus CP.

Pairwise alignments of HelVS-VR21 to the genomes of the viruses appearing in the same clade in the phylogenetic tree revealed decreasing levels of nt identity to: PhlVS (62.32%); GLV (62.04%); LVA (60.62%); PhlVB (60.35%); AtrMoV (60.06%); and CVB (59.56%). These values are slightly higher than the BLAST-based alignments used by PASC (Bao et al., 2014; Supplementary Figure 4).

The ButMV-A and ButMV-B sequences were placed in all phylogenetic trees in a subclade with the type isolate of ButMV (ButMV-J), and also coleus vein necrosis virus (CVNV) and garlic common latent virus (GarCLV), with either 99 or 100% bootstrap support. An additional four viruses formed a larger clade including ButMV in all of the trees, also with either 99% or 100% bootstrap support (Figures 5A–C).

BLASTn analysis of the ButMV-A genome revealed no identities of >70% over the full genome, except for 78.67% nt identity to ButMV-J (AB517596) with 96% coverage, and 81.81% identity to the CP gene of ButMV-Kr-NS5 (MK689358). BLASTp analysis of ButMV-A RdRp showed 100% coverage at 85.93% aa identity to ButMV-J, and no more that 46.17% aa identity to any other viral RdRp. Similar analysis with ButMV-A CP identified 91.22% aa identity with full coverage of the ButMV-J CP.

## Discussion

We report here the identification and near-complete genome sequences of one isolate of HelVS, and two distinct isolates of ButMV, from a mixed infection of an unknown cultivar of *Veronica* sp. (possibly an interspecific hybrid), both first reports for this host genus. We additionally report the sequence of a second isolate of HelVS, from a single infection of a different cultivar of veronica, sold as *Veronica spicata*, or Spike Speedwell.

HelVS was found for the first time in *Veronica* sp. (family Plantaginaceae). This virus has a restricted natural host range and experimental host range, previously reported only from *Helenium amarum* (Asteraceae) in Germany and *Impatiens holstii* (Balsaminaceae) in Minnesota (USA), Yugoslavia, and the Netherlands (Kuschki et al., 1978; Koenig et al., 1983; Pleše et al., 1988). All of the natural hosts reported to date are ornamental plants, suggesting aphid-borne transmission within greenhouses as the means of host range expansion. The mild mottle or mosaic symptoms observed in veronica plants infected solely with HelVS might not be noticed by many nursery growers, which might explain detection in three additional cultivars of *Veronica* sp. by a scientist trained in viruses affecting ornamentals.

We have generated the first near-complete genome sequences of HeLVS, from two distinct sources, from commercial nurseries in Minnesota and Maryland but having a high degree of sequence identity; these are the first HelVS genome sequence beyond 3'-proximal sequences of c.1685 nt from the Helenium isolate (D10454 and S71594), and 935 nt encompassing the 3′-end of the RdRp, and part of TGB1 (FJ555524). The majority of the NGS sequence of HelVS-Ver was further validated by PCR cloning and Sanger sequencing (8,457 of 8,615 nt; Table 2), while 4,138 nt of the 8,668 nt HelVS-V (VR21) was also separately derived by PCR cloning and/or Sanger sequencing, with >99.7% pairwise identity for each NGS/PCR-derived pair.

The established ICTV criteria for distinguishing species with the family *Betaflexiviridae* are that isolates of different species should have less than about 72% nt identity (or 80% aa identity) between their respective CP or polymerase genes. Viruses from different genera usually have less than about 45% nt identity in these genes (ICTV 9th Report, 2011). Phylogenetic trees generated by the Maximum Likelihood (ML) method further demonstrated the close identities between HelVS-V and HelVS-Ver, grouping them into a clade with the same group of other carlavirus species, with high bootstrap support, whether trees were based on the whole genome nt sequence, or the RdRp or CP aa sequences. The highest pairwise genome identity to HeLVS-VR was 62.32% to PhlVS, fully conforming with the ICTV criteria for separating carlavirus species.

PASC analysis (Bao et al., 2014) (Supplementary Figure 4) further confirmed the position of HelVS as a distinct carlavirus sequence, identifying the closest relatives as GLV, PhlVS, and LVA, all of which fell within the same clade as HelVS in all of the phylogenetic trees (Figures 5A–C).

ButMV is also reported for the first time in Veronica, and for the first time in the USA; ButMV has previously been reported only from *Petasites japonicus* (Asteraceae), originally in Japan (Tochihara and Tamura, 1976; Hashimoto et al., 2009), and more recently in Korea (MK689358). It is interesting that both ButMV and HelVS were first reported in species of the Asteraceae, and are now found in mixed infection in a plant belonging to the taxonomically distant Plantaginaceae. Veronica is only the second natural host of ButMV reported, and only the third natural host of HelVS.

The near-complete genome sequences of two distinct isolates were identified from the same plant; isolates ButMV-A and ButMV-B were divergent from each other, and from the type isolate, ButMV-J, but still shared a sufficient degree of identity in their full genomes, and the RdRp and CP amino acid sequences, to clearly be identified as the same carlavirus species as the type isolate, ButMV-J.

Whereas isolate ButMV-A appeared typical of carlaviruses, having the expected complement of six ORFs, encoding RdRp, TGB1, TGB2, TGB3, CP, and NABP, isolate ButMV-B had a major deletion encompassing almost the entire TGB1 ORF; TGB1 is thought to be a significant contributor toward viral movement and replication efficiency, and thus an essential gene. Absence of a functional TGB1 from ButMV-B was confirmed by RT-PCR using different combinations of degenerate primers amplifying both ButMV-A and ButMV-B, and primers specific for ButMV-A or ButMV-B, demonstrating the presence of both types of sequence in the original RNA extract. This was further confirmed by absence of most of the TGB1 ORF in the NGS sequence of ButMV-B. Interestingly, PCR from the original sample using a degenerate primer pair able to amplify across the TGB1 region of ButMV-A resulted in bands of similar intensity for the anticipated products from ButMV-A and ButMV-B, indicating that replication of ButMV-B was not considerably less efficient than for ButMV-A. The relative numbers of reads mapping to ButMV-A (1,561,852) and ButMV-B (1,785,592) sequences obtained from the NGS results from the same RNA sample extract were also quite similar, further suggesting that replication of ButMV-B was not severely debilitated, but comparable to that of ButMV-A.

That ButMV-B is successfully supported by ButMV-A over the long term, rather than being a defective replicon of recent origin, is suggested by the fact that the isolation of the original random PCR RdRp clones and the distinct 3′-terminal ButMV-A and ButMV-B clones were prepared more than 2 years apart, in each case from RNA isolated from fresh tissue. The generation of PCR products across the region of the deletion occurred several months later, so the defective ButMV-B sequence was stably maintained in the presence of ButMV-A for at least 2 years in an actively growing plant.

This appears to be the first case of a defective isolate of any carlavirus, apparently maintained long-term in Veronica as a result of complementation of the missing TGB1 functions by the fully-functional genome of ButMV-A. The essential nature of TGB1 has been demonstrated with the potexviruses white clover mosaic virus (WClMV) and potato virus X (PVX). Lough et al. (1998) showed that an infectious clone of WClMV with a deletion of a portion of TGB1 was still able to replicate in protoplasts, but was not able to spread in whole plants; however, the TGB1 deletion mutant was able to spread in transgenic plants expressing a wild-type TGB1, as a result of functional complementation. Separately, Bayne et al. (2005) generated multiple random mutants in the PVX TGB1, and showed that any mutations defective for RNA silencing suppression were also non-functional for cell-to-cell movement; a number of mutants were also recovered which retained significant RNA silencing suppression activity, but which still lacked movement capability, thus demonstrating that the RNA silencing suppression function is necessary, but not sufficient for cell-to-cell movement.

Distinct isolates of a third potexvirus, Alternanthera mosaic virus, have been shown to co-survive over multiple years and many mechanical transmission passages, interacting to produce an intermediate symptom between the most severe and mildest symptoms induced when individual sequence types were cloned and separated (Lim et al., 2010). This phenomenon was recreated by mixing infectious clones of different symptom severity; one possible advantage of the milder symptoms of the mixed infection might be a lesser effect on the host plant while still allowing high replication levels (Lim et al., 2010).

Whereas there may be a similar advantage to the presumably fully functional ButMV-A, and is clearly an advantage to the defective isolate ButMV-B, the question of the mechanism of origin of ButMV-B is less clear. One possibility is of illegitimate recombination occurring within a formerly wild-type isolate of ButMV resulted in the deletion of essentially the full TGB1 gene, with survival of the mutant due to a mixed infection with ButMV-A (or at least a progenitor sequence). However, it is interesting that the sequence of ButMV-B to the 5′ of the deletion, and the remaining sequence to the 3′ side of the deletion, share different degrees of identity to both ButMV-A and ButMV-J (c.71-72% nt identity for the RdRp region, vs. c.77–78% for the region from TGB2 to the 3′ end) (Table 3). This suggests an alternate possible origin in an illegitimate recombination between two isolates, each distinct from the “helper” isolate ButMV-A, possibly followed by an internal deletion. Either possible explanation suggests that mixed infection of two distinct ButMV isolates is not a recent phenomenon, but has existed for quite some time. Whether such a mixed infection initially occurred in Veronica, in butterbur, or a third and as yet unknown host, and spread to Veronica by aphid transmission is an open question. It is also possible that the presence of a HelVS infection in the same plant also contributes to maintenance of the defective ButMV-B isolate. Certainly, mixed infections and sympatric speciation of multiple carlaviruses have been documented in elderberry (Ho et al., 2016), and other hosts infected by two or more distinct carlaviruses have also been reported (e.g., Van Dijk, 1993; Eastwell and Druffel, 2012; Richert-Pöggeler et al., 2015), suggesting a fertile area for further investigations of interactions between carlavirus species.

## Data Availability Statement

The datasets presented in this study can be found in online repositories. The names of the repository and accession and accession numbers can be found at:

https://www.ncbi.nlm.nih.gov/genbank/, MW207172;

https://www.ncbi.nlm.nih.gov/genbank/, MW207173;

https://www.ncbi.nlm.nih.gov/genbank/, MW207174;

https://www.ncbi.nlm.nih.gov/genbank/, MW225999;

https://www.ncbi.nlm.nih.gov/genbank/, MW226000;

https://www.ncbi.nlm.nih.gov/genbank/, MW226001;

https://www.ncbi.nlm.nih.gov/genbank/, MW226002;

https://www.ncbi.nlm.nih.gov/genbank/, MW226003;

https://www.ncbi.nlm.nih.gov/genbank/, MW226004;

https://www.ncbi.nlm.nih.gov/genbank/, MW246808;

https://www.ncbi.nlm.nih.gov/genbank/, MW246809;

https://www.ncbi.nlm.nih.gov/genbank/, MW246810;

https://www.ncbi.nlm.nih.gov/genbank/, MW246811;

https://www.ncbi.nlm.nih.gov/genbank/, MW246812.

## Author Contributions

JH and DM developed and directed the study. JH, DM, and RJ supervised the next-generation sequencing. MR, RJ, and SG performed the NGS analyses. BL and DM performed the initial extraction and random PCR cloning. MR performed most of the PCR and cloning. JH performed additional sequence analyses and wrote the first draft, with assistance from RJ and DM and input from MR and SG. All authors assisted in revisions and approved the manuscript for publication.

## Conflict of Interest

The authors declare that the research was conducted in the absence of any commercial or financial relationships that could be construed as a potential conflict of interest.

## Supplementary Material

Supplementary Figure 1 Graphical representation of a carlavirus genome and the encoded proteins, with approximate sizes and positions of PCR products derived from the initial 3′-proximal regions, and the random-PCR products from the RdRp region, aligned and colored to indicate the virus from which each originates, with ButMV-related products in blue, and HelVS-related products in red. The approximate overlaps of RdRp-d and RdRp-b, and of RdRp-b and HelVS-I (GenBank acc. no. FJ555524) are also shown. The 3′-proximal PCR products and associated random PCR RdRp products are shown in line with each other (see also Table 1).

Supplementary Figure 2 Genome coverage of the Helenium virus S genome by the next-generation sequencing reads, which averaged 14,493 reads per nucleotide.

Supplementary Figure 3 Graphical illustration of the strategy for PCR amplification and Sanger sequencing of a series of overlapping PCR product covering 8,457 nt of the 8,615 nt HelVS-Ver genome obtained by next-generation sequencing.

Supplementary Figure 4 Pairwise sequence comparison (PASC) (Bao et al., 2014) result from analysis at the NCBI website, showing that HelVS is distinct from other characterized carlavirus species.

Supplementary Table 1 Primers utilized in this study; unless otherwise annoted, all primers were designed for this study.
