# Supplementary material for: A Mixed Infection of Helenium Virus S With Two Distinct Isolates of Butterbur Mosaic Virus, One of Which Has a Major Deletion in an Essential Gene
Source: Front Microbiol. 2020 Dec 21;11:612936. doi: 10.3389/fmicb.2020.612936 (PMC7779399; doi:10.3389/fmicb.2020.612936)
Supplement: Supplementary Figure 1 — Graphical representation of a carlavirus genome and the encoded proteins, with approximate sizes and positions of PCR products derived from the initial 3′-proximal regions, and the random-PCR products from the RdRp region, aligned and colored to indicate the virus from which each originates, with ButMV-related products in blue, and HelVS-related products in red. The approximate overlaps of RdRp-d and RdRp-b, and of RdRp-b and HelVS-I (GenBank acc. no. FJ555524) are also shown. The 3′-proximal PCR products and associated random PCR RdRp products are shown in line with each other (see also Table 1). [file Data_Sheet_1.zip › Supplemental files/Supplementary Figure Legends.docx]

**SUPPLEMENTARY FIGURE LEGENDS**

**Supplementary Figure 1.** Graphical representation of a carlavirus genome and the encoded proteins, with approximates sizes and positions of PCR products derived from the initial 3ʹ-proximal regions, and the random-PCR products from the RdRp region, aligned and colored to indicate the virus from which each originates, with ButMV-related products in blue, and HelVS-related products in red. The approximate overlaps of RdRp-d and RdRp-b, and of RdRp-b and HelVS-I (GenBank acc. no. FJ555524) are also shown. The 3ʹ-proximal PCR products and associated random PCR RdRp products are shown in line with each other (see also Table 1).

**Supplementary Figure 2.** Genome coverage of the Helenium virus S genome by the next-generation sequencing reads, which averaged 14,493 reads per nucleotide.

**Supplementary Figure 3.** Graphical illustration of the strategy for PCR amplification and Sanger sequencing of a series of overlapping PCR product covering 8,457 nt of the 8,615 nt HelVS-Ver genome obtained by next-generation sequencing.

**Supplementary Figure 4.** Pairwise sequence comparison (PASC) (Bao et al., 2014) result from analysis at the NCBI website, showing that HelVS is distinct from other characterized carlavirus species.
