# Supplementary material for: A Mixed Infection of Helenium Virus S With Two Distinct Isolates of Butterbur Mosaic Virus, One of Which Has a Major Deletion in an Essential Gene
Source: Front Microbiol. 2020 Dec 21;11:612936. doi: 10.3389/fmicb.2020.612936 (PMC7779399; doi:10.3389/fmicb.2020.612936)
Supplement: Supplementary Figure 1 — Graphical representation of a carlavirus genome and the encoded proteins, with approximate sizes and positions of PCR products derived from the initial 3′-proximal regions, and the random-PCR products from the RdRp region, aligned and colored to indicate the virus from which each originates, with ButMV-related products in blue, and HelVS-related products in red. The approximate overlaps of RdRp-d and RdRp-b, and of RdRp-b and HelVS-I (GenBank acc. no. FJ555524) are also shown. The 3′-proximal PCR products and associated random PCR RdRp products are shown in line with each other (see also Table 1). [file Data_Sheet_1.zip › Supplemental files/Supplementary Table 1 - primers.docx]

**Supplementary Table 1**. Primers utilized in this study; unless otherwise annotated, all primers were designed for this study.

| **Primer name** | **Primer Sequence** | **Purpose** |
| --- | --- | --- |
| NSNC-odT | 5ʹ-ATCCATGGCATGCATCGATTTTTTTTTTTTTTTV-3ʹ | cDNA production ^a^ |
| BNSNC | 5ʹ-TTTATCGGATCCATGGCATGCATCG-3ʹ | 3ʹ-reverse primer ^a^ |
| PxDeg | 5ʹ-GGNGGNNVNTAYNIIGAYGGIACIAAR-3ʹ | Forward primer in TGB2 ^b^ |
| ButRepFdeg | 5ʹ-GACAATTAYGCMATAGAGGTTTCC-3ʹ | Amplify across TGB1 gene |
| ButTGBRdeg | 5ʹ- TAAGAKGTRTGACYGCATCTATG-3ʹ |  |
| ButA-TGB | 5ʹ-CACACAAATATTGTTACCTAGCTTG-3ʹ | ButMV-A specific reverse primer |
| ButB-TGB | 5ʹ-CACACAAACACTATTATTGGATCCT-3ʹ | ButMV-B specific reverse primer |
| VerRdRpD-F1 | 5ʹ-TACAGGGGTCGGTGCCTTGG-3ʹ | To sequence RdRp-d to 3ʹ HelVS sequence |
| VerRdRpD-F2 | 5ʹ-CAGTTTGTGTCAGGCCTACAGG-3ʹ | To link RdRp-d to 3ʹ HelVS sequence |
| HVS-rep4 | 5ʹ-TTTGAATAAAGGTGAGCGAT-3ʹ | To sequence 3ʹ HelVS sequence to RdRp-d |
|  |  |  |
| VerF1 | 5ʹ-AGGGAGGAGGGAAGAAGTTG-3ʹ | 3ʹ HelVS-Ver PCR genome product A |
| BNSNC | 5ʹ-TTTATCGGATCCATGGCATGCATCG-3ʹ |  |
| HVS-rep1 | 5ʹ-CTAATAAACGCTGATTCGC-3ʹ | HelVS-Ver PCR genome product B |
| VerR1 | 5ʹ- TCCTGAACCTTGCCCATAAC-3ʹ |  |
| HVS-rep6 | 5ʹ- TGACATTCTTTATGGCTGTG-3ʹ | HelVS-Ver PCR genome product C |
| HVS-rep2 | 5ʹ- CGAAGTTACGAATGTAACGG-3ʹ |  |
| HVS-rep9 | 5ʹ-TGTCACCCCGTAGAGCATTG-3ʹ | HelVS-Ver PCR genome product D |
| HVS-rep10 | 5ʹ-AACTTCCCGTAGCAAGAGGC-3ʹ |  |
| HVS-rep11 | 5ʹ-AGCAGTTGGAGTCACACAGG-3ʹ | HelVS-Ver PCR genome product E |
| HVS-rep12 | 5ʹ-CCAATTTCCCGGCACCTTTC-3ʹ |  |
| HVS-rep13 | 5ʹ-CCCTATGCTGAACTGCCCAA-3ʹ | HelVS-Ver PCR genome product F |
| HVS-rep14 | 5ʹ-TCCACCTCCCTTCCATCAGT-3ʹ |  |
| HVS-rep15 | 5ʹ-CGCCAAACAGCATCTCATCG-3ʹ | HelVS-Ver PCR genome product G |
| HVS-rep16 | 5ʹ-GGCAACTTGCGAGCAATGTA-3ʹ |  |

^a^ These primers were previously developed; see Hammond et al. (2005; Identification and full sequence of an isolate of Alternanthera mosaic potexvirus infecting *Phlox stolonifera*. Arch. Virol. 151:477-493).

^b^ Primer PxDeg was also previously developed; see Hammond and Reinsel (2011; Mixed infections and novel viruses in various species of Phlox. Acta Hortic. 901:119-126).
