# Supplementary figures and images for: A Mixed Infection of Helenium Virus S With Two Distinct Isolates of Butterbur Mosaic Virus, One of Which Has a Major Deletion in an Essential Gene
Source: Front Microbiol. 2020 Dec 21;11:612936. doi: 10.3389/fmicb.2020.612936 (PMC7779399; doi:10.3389/fmicb.2020.612936)

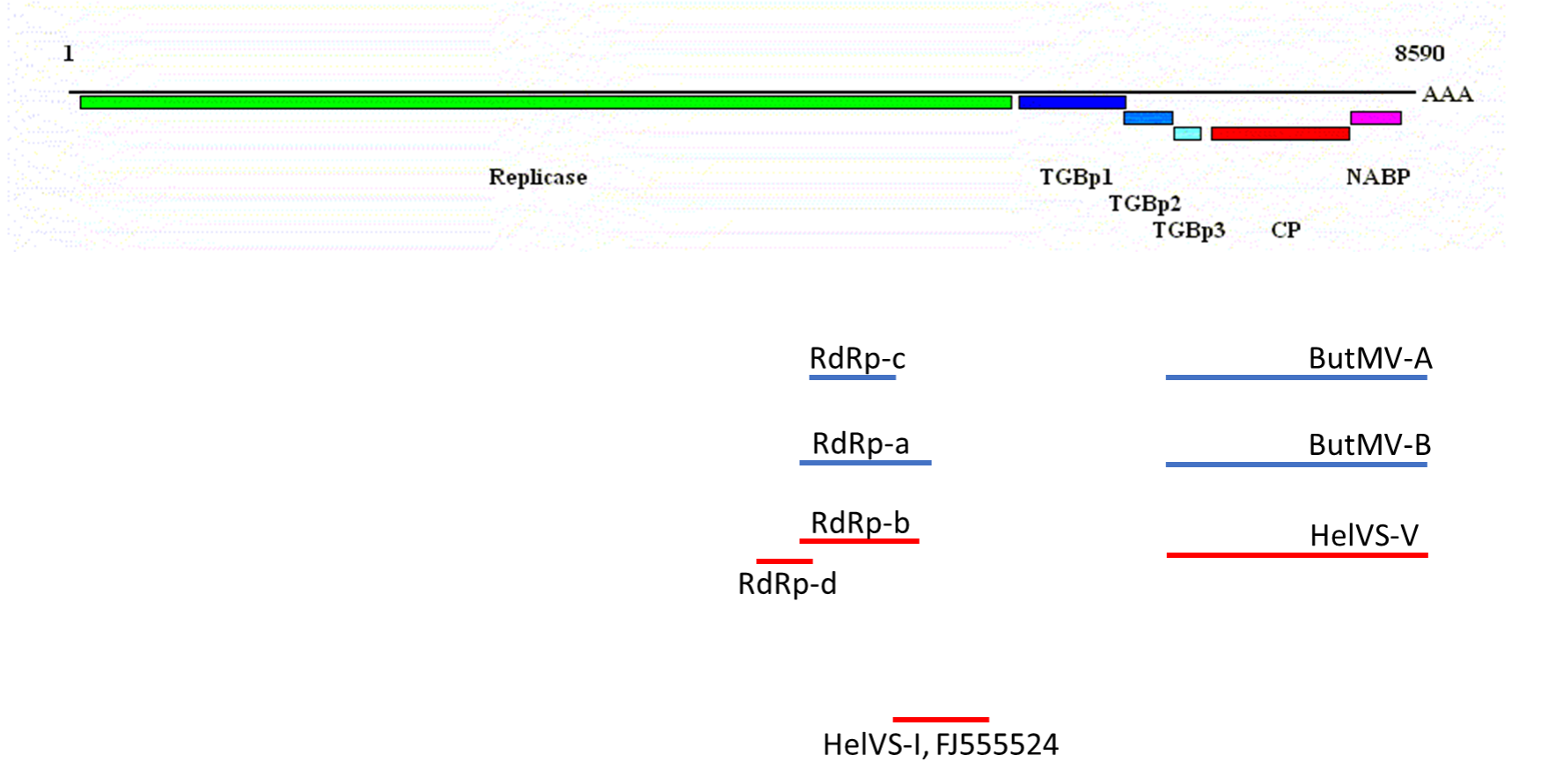

Supplement: Supplementary Figure 1 — Graphical representation of a carlavirus genome and the encoded proteins, with approximate sizes and positions of PCR products derived from the initial 3′-proximal regions, and the random-PCR products from the RdRp region, aligned and colored to indicate the virus from which each originates, with ButMV-related products in blue, and HelVS-related products in red. The approximate overlaps of RdRp-d and RdRp-b, and of RdRp-b and HelVS-I (GenBank acc. no. FJ555524) are also shown. The 3′-proximal PCR products and associated random PCR RdRp products are shown in line with each other (see also Table 1). [file Data_Sheet_1.zip › Supplemental files/Supplementary Figure 1.png]

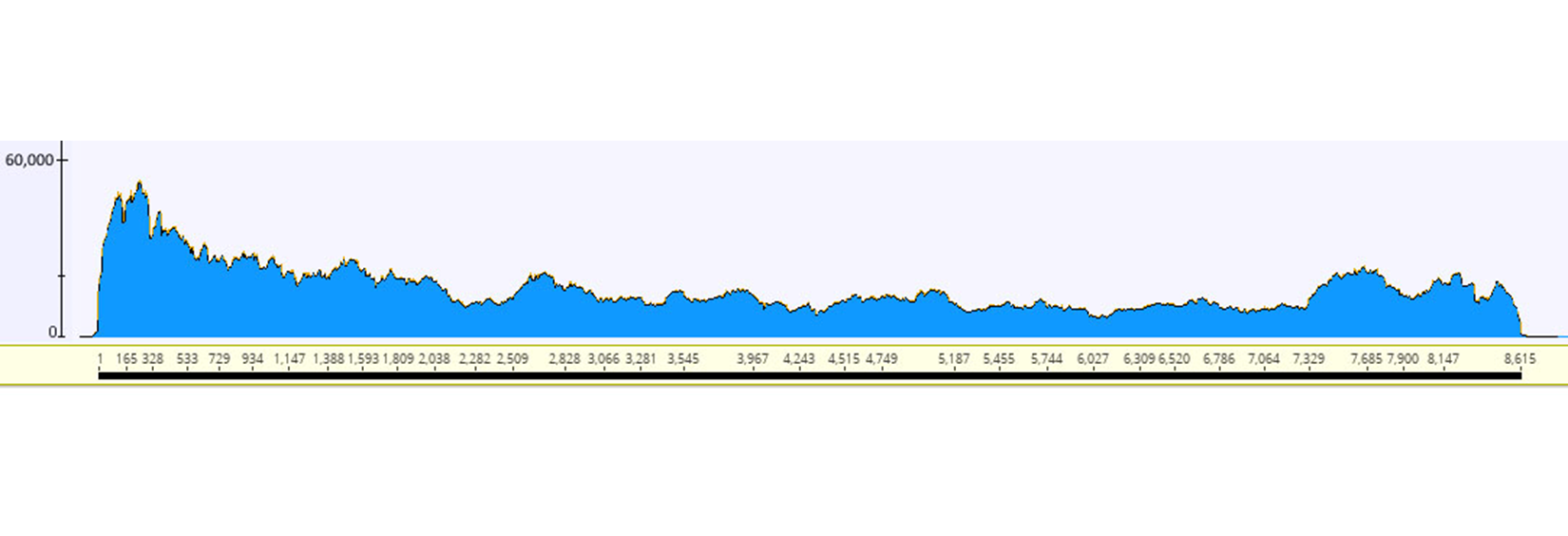

Supplement: Supplementary Figure 1 — Graphical representation of a carlavirus genome and the encoded proteins, with approximate sizes and positions of PCR products derived from the initial 3′-proximal regions, and the random-PCR products from the RdRp region, aligned and colored to indicate the virus from which each originates, with ButMV-related products in blue, and HelVS-related products in red. The approximate overlaps of RdRp-d and RdRp-b, and of RdRp-b and HelVS-I (GenBank acc. no. FJ555524) are also shown. The 3′-proximal PCR products and associated random PCR RdRp products are shown in line with each other (see also Table 1). [file Data_Sheet_1.zip › Supplemental files/Supplementary Figure 2.png]

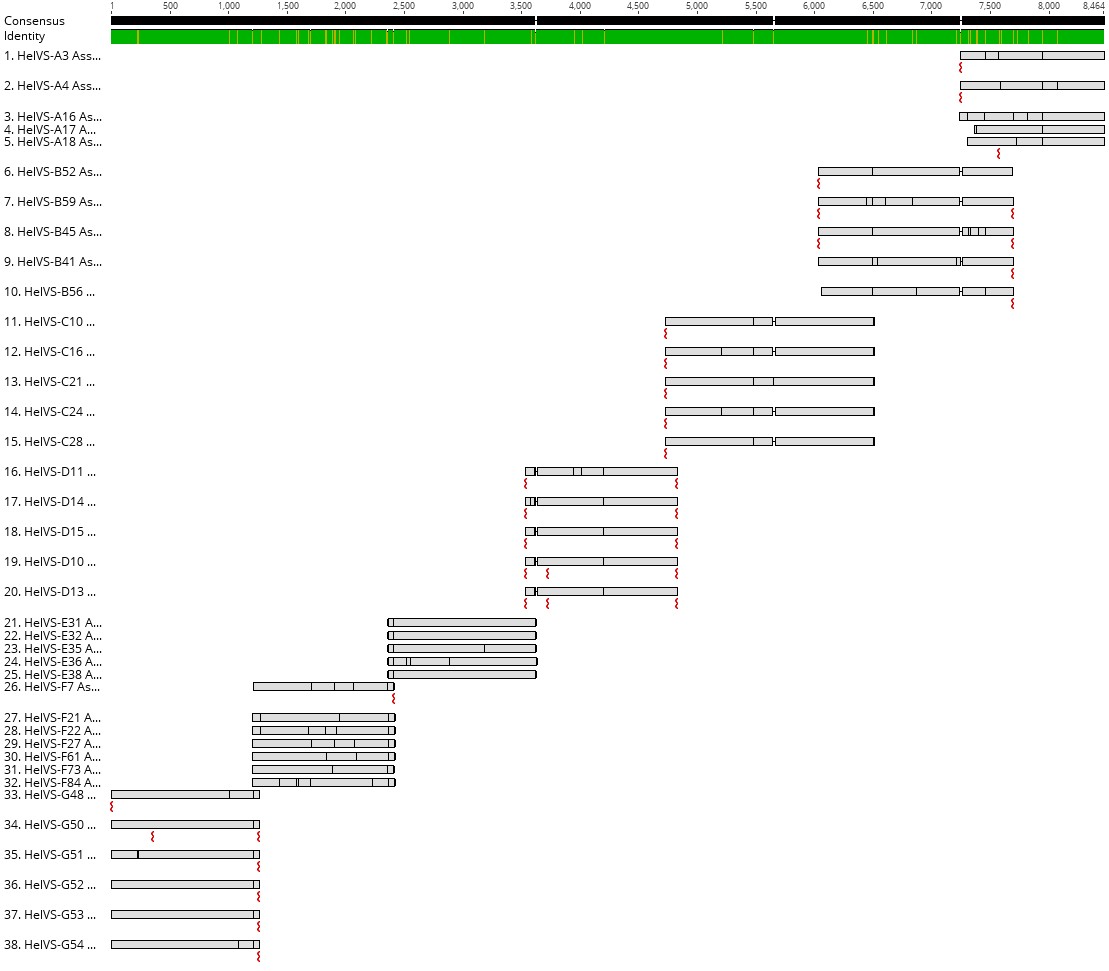

Supplement: Supplementary Figure 1 — Graphical representation of a carlavirus genome and the encoded proteins, with approximate sizes and positions of PCR products derived from the initial 3′-proximal regions, and the random-PCR products from the RdRp region, aligned and colored to indicate the virus from which each originates, with ButMV-related products in blue, and HelVS-related products in red. The approximate overlaps of RdRp-d and RdRp-b, and of RdRp-b and HelVS-I (GenBank acc. no. FJ555524) are also shown. The 3′-proximal PCR products and associated random PCR RdRp products are shown in line with each other (see also Table 1). [file Data_Sheet_1.zip › Supplemental files/Supplementary Figure 3.jpg]

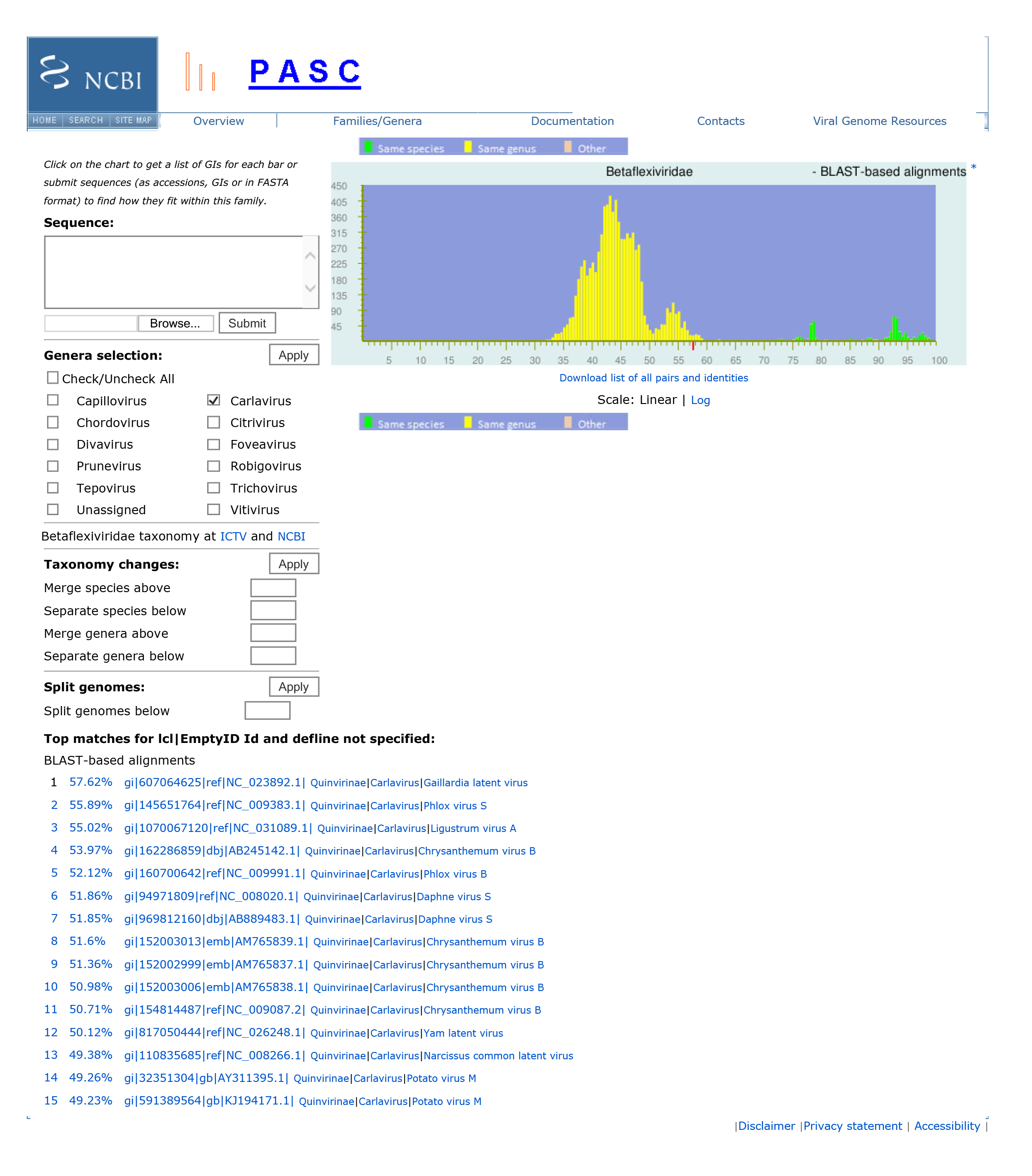

Supplement: Supplementary Figure 1 — Graphical representation of a carlavirus genome and the encoded proteins, with approximate sizes and positions of PCR products derived from the initial 3′-proximal regions, and the random-PCR products from the RdRp region, aligned and colored to indicate the virus from which each originates, with ButMV-related products in blue, and HelVS-related products in red. The approximate overlaps of RdRp-d and RdRp-b, and of RdRp-b and HelVS-I (GenBank acc. no. FJ555524) are also shown. The 3′-proximal PCR products and associated random PCR RdRp products are shown in line with each other (see also Table 1). [file Data_Sheet_1.zip › Supplemental files/Supplementary Figure 4.tif]
